# Association between chronic kidney disease and tooth loss among Korean adults: results from the Korea National Health And Nutrition Examination Survey (KNHANES), 2013–2018

**DOI:** 10.1080/0886022X.2025.2531239

**Published:** 2025-07-20

**Authors:** Na-Yeong Kim, Ki-Ho Chung

**Affiliations:** a Department of Preventive and Public Health Dentistry, Chonnam National University School of Dentistry, Gwangju, Republic of Korea; b Dental Science Research Institute, Chonnam National University, Gwangju, Republic of Korea

**Keywords:** Chronic kidney disease, estimated glomerular filtration rate, tooth loss, 2021 CKD-EPI, KNHANES

## Abstract

Chronic kidney disease (CKD) and oral health are important public health problems worldwide, resulting in various complications. This study aimed to confirm the association between CKD and the number of teeth using data from the Korea National Health and Nutrition Examination Survey (KNHANES), which is representative of Korean adults. This study used raw data from the 6th and 7th (2013–2018) KNHANES and targeted 16,125 adults aged 40 years or older in Korea. Chronic kidney disease was defined as 2021 Chronic Kidney Disease Epidemiology Collaboration (CKD-EPI). Multiple logistic regression analysis was used to examine the association between CKD and the number of teeth. The prevalence of CKD was significantly higher in the group with fewer than 20 teeth. In the multivariate logistic regression analysis, CKD was associated with having fewer than 20 teeth after adjusting for age, sex, household income, education, alcohol consumption, smoking, body mass index, hypertension, diabetes mellitus, angina, myocardial infarction, stroke, dyslipidemia, and performance of an oral examination within 1 year, daily toothbrushing frequency, and hygiene product use (Odds ratio = 1.34; 95% Confidence Interval = 1.03–1.74). Therefore, CKD may contribute to an increased risk of tooth loss. Implementing an integrated healthcare approach for oral health management in individuals with CKD could help reduce their burden of oral diseases.

## Graphical abstract

**Figure:**
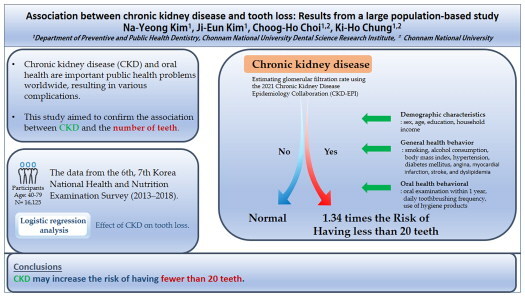

## 1. Introduction

Chronic kidney disease (CKD) is a progressive disease characterized by structural and functional changes in the kidney over several months or years due to various causes and is a global public health problem [1,2]. The prevalence of CKD is predicted to increase rapidly by 2040, causing it to become the fifth leading cause of death worldwide [3]. Diabetes and hypertension are typical causes of CKD, and complications, such as anemia, bone disease, cardiovascular disease, and an increased risk of cancer, may appear [4,5]. Previous studies have shown that 90% of patients with chronic renal failure (CRF) have oral lesions that affect both bone and soft tissue [6].

International guidelines define CKD as decreased renal function with a glomerular filtration rate (GFR) of less than 60 mL/min per 1.73 m^2^ [4]. The GFR is widely accepted as the best overall measure of kidney function in health and disease [7]. A variety of simple equations for determining the estimated GFR (eGFR) are widely available [8,9]. Among them, the Modification of Diet in Renal Disease (MDRD) equation and the Chronic Kidney Disease Epidemiology Collaboration (CKD-EPI) equation are used internationally [10]. Recently, the National Kidney Foundation (NKF) and the American Society of Nephrology (ASN) formed a task force to reevaluate the inclusion of race in kidney disease diagnosis [11]. The final report recommended the 2021 CKD-EPI creatinine and creatinine-cystatin C equations adjusted for age and sex and unadjusted for race [11].

Oral disease is one of the most prevalent diseases worldwide, and dental caries, periodontal disease, and tooth loss are the most common oral health-related disease. Poor oral health is also associated with poor oral health-related quality of life (OHRQoL) [12]. In particular, tooth loss is one of the main indicators of adult oral health and is related to various factors such as race and socioeconomic status imbalance [13]. Previous longitudinal studies have reported an association between tooth loss, reduced occlusal function, and bite problems in older adults [14,15]. In addition, a decrease in the number of teeth is associated with the risk of cognitive decline, and the number of deaths due to cognitive decline and oral health is gradually increasing [16]. These findings suggest that oral health is increasingly important due to the burden of tooth loss and that tooth loss is an important indicator of life.

To date, some domestic and foreign precedent studies on the association between CKD and the number of teeth have been conducted, but they are still insufficient. A study by Yoshihara et al. [17] reported a significant association between the number of teeth and serum cystatin C levels in elderly Japanese women, but the number of participants was rather small (∼600 subjects). Parente et al. [18] reported a higher prevalence of edentulousness among individuals with terminal chronic diseases compared to healthy controls. These studies have focused on associations in subgroups, such as the elderly, and differed in how they assessed kidney function. Although the 2021 CKD-EPI creatinine equation is currently recommended, studies analyzing the association between CKD, defined by using this equation and the number of teeth have not been conducted to date.

Therefore, this study aimed to analyze the association between CKD and the number of teeth in Korean adults aged 40 years and older using the GFR estimated by the latest equation, the 2021 CKD-EPI creatinine equation.

## 2. Materials and methods

### 2.1. Research participants and data

**Figure 1.**
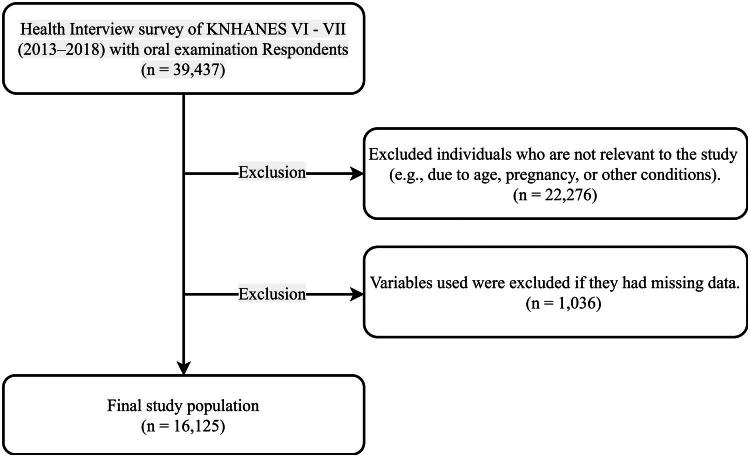
Composition of the final study population sample from the total number of participants.

This study used raw data from the 6th and 7th (2013–2018) Korea National Health and Nutrition Examination Survey (KNHANES) conducted by the Ministry of Health and Welfare and the Korea Disease Control and Prevention Agency. To improve the representativeness of the sample and the accuracy of the estimates, KNHANES used a two-stage stratified cluster sampling method with 192 sampling sites per year and 3,840 households in the sixth period (2013–2015) and 4,416 households in the seventh period (2016–2018) as the primary and secondary sampling units to select all eligible household members aged 1 year and above within the sampled households. KNHANES has three parts: health interview, health examination, and nutritional survey. The health interview covers morbidity, healthcare utilization, education, and economic activity as well as smoking, drinking, mental health, and oral health. The health examination consists of physical measurements (e.g. blood pressure and pulse), blood and urine tests, and an oral examination. The data used in the study were approved by the Research Ethics Review Committee of the Korea Disease Control and Prevention Agency (2013-07CON-03-4C, 2013-12EXP-03-5C, 2018-01-03-P-A). All participants signed an informed consent form prior to participation. The total number of participants in the 6th and 7th waves of the study (2013–2018) was 39,437. Among adults between the ages of 40 and 79 years, women who were pregnant or breastfeeding after childbirth and those with missing values among the study variables were excluded. Ultimately, 16,125 participants were selected as the final participants (Figure 1).

### 2.2. Demographic characteristics

The covariates used in this study were modified and supplemented from those used in previous studies [19]. Age, education status, and household income were selected as demographic variables. Age was classified into 40–65 years and 66–79 years based on the life transition period. Education status was classified into elementary school graduation or lower, middle school graduation, high school graduation, and college graduation or higher based on the school of graduation. Finally, household income was divided into lowest, second lowest, second highest, and highest based on the quartile of household income.

### 2.3. General health behavior

Smoking was classified as current, past, or nonsmoking, and drinking was classified into low or high according to the frequency of intake. BMI was calculated using the weight (kg)/height^2^ (m)^2^ formula. BMI was classified into <18.5 kg/m^2^, 18.5–25.0 kg/m^2^, and ≥25 kg/m^2^. Blood pressure was measured three times after resting in a sitting position for five minutes, and the average value of the second and third measurements was used for analysis. Hypertension was defined as a systolic blood pressure greater than 140 mmHg or a diastolic blood pressure greater than 90 mmHg or taking antihypertensive medication; pre-hypertension was defined as a systolic blood pressure greater than 120 mmHg but less than 140 mmHg or a diastolic blood pressure greater than 80 mm Hg but less than 90 mmHg; and the rest were classified as normal. Blood samples were collected from each participant antecubital vein after fasting for at least eight hours. Blood samples were analyzed within 24 h of transport. Diabetes mellitus was defined as having a fasting blood glucose level of 126 mg/dL or higher, having a physician diagnosis, taking blood glucose-lowering medication, or receiving insulin injections; impaired fasting glucose was defined as a fasting blood glucose between 100 and 125 mg/dL; and normal was defined as a fasting blood glucose less than 100 mg/dL. Angina, myocardial infarction, stroke, and dyslipidemia were categorized as present or absent based on physician diagnosis.

### 2.4. Oral health behavior

The performance of an oral examination was classified as yes or no depending on whether or not the participants underwent an oral examination in the past year. The daily toothbrushing frequency was classified as more than three times or less than three times. Finally, hygiene product use was defined as the use of either an interdental brush or dental floss. If these tools were not used, it was classified as nonuse.

### 2.5. CKD

CKD was defined as CKD when the eGFR <60 mL/min/1.73 m^2^ after calculating eGFR using the 2021 CKD-EPI creatinine equation [11]. The 2021 KD-EPI creatinine equation is as follows: 142 × min (S_cr_/κ,1)^α^ × max (S_cr_/κ,1)^−1.200^ × 0.9938^age^ × β (S_cr_: serum creatinine; κ: 0.7 for women, 0.9 for men; α: −0.241 for women, −0.302 for men; β: 1.012 for women, 1 for men; min: minimum of S_cr_/κ or 1; max: maximum of S_cr_/κ or 1).

### 2.6. Number of teeth

For the number of teeth, a total of 28 teeth excluding the third molar were added. A previous study suggested that 20 teeth were required for minimum masticatory ability [20]. The number of teeth was classified as <20 or ≥20.

### 2.7. Statistical analysis

Since the KNHANES is a complex sample design, analysis of complex sample data was conducted in consideration of strata, cluster, and weight. The Chi-square test and t-test were conducted to verify the difference in CKD prevalence according to demographic characteristics, general health behavior, oral health behavior, CKD status, and number of teeth of the survey participants. Data were expressed as the mean ± standard error (SE) or frequency (%). To investigate the effect of CKD and confounding factors on the number of teeth, a multivariate regression model was constructed, and odds ratios (ORs) and 95% confidence intervals (CIs) were calculated through multivariate logistic regression analysis. Logistic regression analysis was performed to determine the effect of risk factors (age, smoking, hypertension, diabetes) on the association between tooth loss and CKD. All analyses were performed using SAS 9.4 software (SAS Institute, Inc., Cary, NC, USA), and complex sample procedures such as PROC SURVEYLOGISTIC were used to account for the complex survey design. The statistical significance level was set at *p* < 0.05.

## 3. Results

### 3.1. General characteristics

General characteristics according to the sex of the participants were analyzed (Table 1). A total of 16,125 participants were studied; 428 (2.7%) had CKD, and 15,697 (97.3%) did not. All confounding factors were significantly different according to the presence or absence of CKD (*p* < 0.05). The average number of teeth was 20.8 ± 0.4 for participants with CKD and 24.4 ± 0.4 for participants without CKD.

**Table 1.** General characteristics of research participants according to the presence or absence of CKD.

Variables	Total	CKD		*P*
		No	Yes	
	(*n* = 16,125)	(*n* = 15,697)	(*n* = 428)	
Age (years)				<0.001
mean ± SE	55.6 ± 0.1	55.3 ± 0.6	67.6 ± 0.6	<0.001
40–65	11,950 (80.2)	11,827 (81.3)	123 (36.0)	
66–79	4,175 (19.8)	2,870 (18.7)	305 (64.0)	
Household income				<0.001
Lowest	3,200 (17.4)	3,017 (16.9)	183 (39.4)	
Second lowest	4,076 (24.6)	3,966 (24.5)	110 (26.3)	
Second highest	4,195 (27.2)	4,123 (27.5)	72 (16.3)	
Highest	4,654 (30.8)	4,591 (31.2)	63 (18.1)	
Education				<0.001
≤Elementary school	4,221 (22.8)	4,014 (22.3)	207 (45.4)	
Middle school	2,224 (13.6)	2,165 (13.6)	59 (14.3)	
High school	5,215 (34.9)	5,107 (35.1)	108 (26.1)	
≥College	4,465 (28.7)	4,411 (29.1)	54 (14.2)	
Alcohol consumption				0.019
High	3,514 (23.5)	3,440 (23.7)	74 (17.8)	
Low	12,611 (76.5)	12,257 (76.3)	354 (82.2)	
Smoking				0.001
Current	2,690 (19.1)	2,625 (19.2)	65 (14.0)	
Past	3,644 (23.0)	3,508 (22.8)	136 (31.9)	
Nonsmoking	9,791 (57.9)	9,564 (57.9)	227 (54.1)	
Body mass index (kg/m^2^)				0.001
≥25	5,815 (35.4)	5,615 (36.2)	200 (45.8)	
18.5–25	9,936 (62.3)	9,716 (62.5)	220 (52.4)	
<18.5	374 (2.3)	366 (2.3)	8 (1.8)	
Hypertension				<0.001
Hypertension	6,179 (35.4)	5,842 (34.5)	337 (78.1)	
Pre-hypertension	4,080 (25.6)	4,046 (26.0)	34 (6.2)	
Normal	5,866 (39.0)	5,809 (39.5)	57 (15.6)	
Diabetes mellitus				<0.001
Diabetes mellitus	2,452 (13.8)	2,271 (13.1)	185 (41.7)	
Impaired fasting glucose	4,505 (28.0)	4,412 (28.2)	93 (22.5)	
Normal	9,164 (58.2)	9,014 (58.7)	150 (35.8)	
Oral exam within 1 year				0.001
Yes	5,636 (34.5)	5,527 (34.7)	109 (25.0)	
No	10,489 (65.5)	10,170 (65.3)	319 (75.0)	
Daily toothbrushing frequency				<0.001
≥3	7,909 (49.8)	7,770 (50.2)	139 (33.3)	
<3	8,216 (50.2)	7,927 (49.8)	289 (66.7)	
Hygiene product use				<0.001
Use	5,214 (33.6)	5,142 (34.0)	72 (18.6)	
Nonuse	10,911 (66.4)	10,555 (66.0)	356 (81.4)	
Number of teeth				<0.001
mean ± SE	24.3 ± 0.1	24.4 ± 0.4	20.8 ± 0.4	<0.001
≥20	2,565 (13.9)	13,294 (86.6)	266 (64.4)	
<20	13,560 (86.1)	2,403 (13.4)	162 (35.6)	
Angina				<0.001
Yes	389 (2.1)	351 (1.9)	38 (8.8)	
No	15,736 (97.9)	15,346 (98.1)	390 (91.2	
myocardial infarction				<0.001
Yes	191 (1.0)	167 (0.9)	24 (6.2)	
No	15,934 (99.0)	15,530 (99.1)	404 (93.8)	
Stroke				<0.001
Yes	448 (2.5)	402 (2.3)	46 (10.6)	
No	15,677 (97.5)	15,295 (97.7)	382 (89.4)	
Dyslipidemia				<0.001
Yes	3,530 (19.4)	3,366 (19.0)	164 (35.3)	
No	12,595 (80.6)	12,331 (81.0)	264 (64.7)	
eGFR (mL/min/1.73 m^2^)	94.5 ± 0.2	95.5 ± 0.9	48.0 ± 0.9	<0.001

All values represent the mean ± SE or numbers and frequencies [N (weighted %)].

### 3.2. CKD and number of teeth

The eGFR and prevalence of CKD according to the number of teeth of the study participants were confirmed (Table 2). The average eGFR of all participants was lower for those with fewer than 20 teeth than for those with more than 20 teeth (89.3 ± 0.4 vs. 95.6 ± 0.2). The prevalence of CKD in all participants was 2.3%, and it was 6.3% when the number of teeth was fewer than 20.

**Table 2.** Prevalence of CKD by the number of teeth.

Variables	Total	Number of teeth		P
	(*n* = 16,125)	<20	≥20	
eGFR (mL/min/1.73 m^2^)				
Total	94.5 ± 0.2	89.3 ± 0.4	95.6 ± 0.2	<0.001
CKD^a^				
Yes	428 (2.3)	162 (6.3)	266 (2.0)	0.002
No	15,697 (97.7)	2,406 (93.7)	13,294 (98.0)	

All values represent the mean ± SE or numbers and frequencies [N (weighted %)].

^a^ CKD is defined when the eGFR is <60 mL/min/1.73 m^2^.

The effect of CKD on a number of teeth of fewer than 20 was analyzed (Table 3). In Model 4, which was adjusted for all confounding factors, the association between CKD and fewer than 20 teeth was significant (OR = 1.34, 95% CI: 1.03–1.74).

**Table 3.** Effect of CKD on a number of teeth of fewer than 20.

Variables	Classification	OR (95% CI)			
		Model 1	Model 2	Model 3	Model 4
CKD	No	1	1	1	1
	Yes	3.58 (2.83–4.53)	1.59 (1.23–2.06)	1.36 (1.05–1.76)	1.34 (1.03–1.74)

OR: odds ratio; CI: confidence interval.

Model 1 was an unadjusted model.

Model 2 was adjusted for age and sex.

Model 3 was adjusted for all the variables in model 2 and household income, education, alcohol consumption, smoking, BMI, hypertension, diabetes mellitus, angina, myocardial infarction, stroke, and dyslipidemia.

Model 4 was adjusted for all the variables in model 3 and oral exam within 1 year, daily toothbrushing frequency, and hygiene product use.

**Figure 2.**
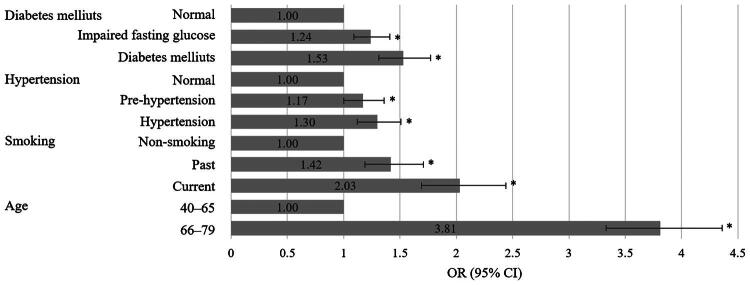
Effect of risk factors on association between tooth loss and CKD. OR: odds ratio; CI: confidence interval. Adjusted for sex, age, household income, education, alcohol consumption, smoking, BMI, hypertension, diabetes mellitus, angina, myocardial infarction, stroke, dyslipidemia, oral exam within 1 year, daily tooth brushing frequency, and hygiene product use (*P < 0.05)

We analyzed the effect of having fewer than 20 teeth on the potential confounders between tooth loss and CKD (Figure 2). Age, smoking, hypertension, and diabetes significantly increased the risk of tooth loss (*p* < 0.05, OR = 3.81, 95% CI: 3.33–4.36; OR = 2.03, 95% CI: 1.69–2.44; OR = 1.30, 95% CI: 1.12–1.51; OR = 1.53, 95% CI: 1.31–1.77, respectively).

## Discussion

This study confirmed an association between CKD and the number of teeth using data from the 6th and 7th KNHANES. There has been no previous study that has classified CKD with the 2021 CKD-EPI creatinine equation and analyzed the association with oral health status.

As a result of this study, participants with CKD had fewer teeth on average than participants without CKD. In addition, the prevalence of CKD was higher in the group with fewer than 20 teeth. Furthermore, in a study of adults over 40 years of age in the United States, 17% of individuals with CKD were found to be edentulous [21]. According to these results, individuals with CKD with reduced renal function may have a reduced number of teeth and a higher likelihood of subsequent edentulousness.

Compared with those without CKD, those with CKD had a 1.36-times increased risk of having fewer than 20 teeth after adjusting for confounding factors (*p* < 0.05). This finding is similar to those of previous studies reporting the relationship between decreased renal function and tooth loss ^17^. Based on the results of this study, the oral health condition of the participants was appropriately reflected by the number of teeth because the teeth were classified according to the criteria of Peres et al. [20] who considered 20 teeth necessary for minimum masticatory function.

The mechanism by which CKD increases the risk of a decreased number of teeth remains unclear. A possible mechanism is that CKD and periodontal disease, which is a major cause of tooth loss, share proinflammatory markers. The association between various inflammatory markers and CKD is currently being reported [22]. Inflammation can be seen as an intermediate factor between oral health behaviors and systemic diseases, and poor oral health behaviors can exacerbate inflammation. People with a low eGFR had higher levels of proinflammatory mediators including IL-1β, IL-6, TNF-α, and hs-CRP [23], and higher levels of hs-CRP accelerated the occurrence of CKD [24]. In patients receiving hemodialysis, inflammatory cytokines and serum CRP levels were associated with periodontal [25]. This evidence suggests that inflammation may play an important role in linking CKD and the number of teeth.

In addition, tooth loss and CKD have complex interactions that can cause issues such as mineral bone disorders, acidosis, and altered oral flora [26]. Decreased kidney function can lead to structural problems such as impaired bone remodeling [27]. CKD is also associated with impaired vitamin D metabolism, potentially giving rise to immune problems and adverse effects on the alveolar bone [26,28]. CKD can lead to metabolic acidosis, involving a decrease in systemic pH, which can reduce bone mineral content and quality while the bone buffers the accumulated acid [29]. In addition, CKD patients on hemodialysis commonly suffer from dry mouth and dysgeusia, poor gingival and periodontal health, and a higher mean Decayed, Missing, and Filled Teeth (DMFT) [30,31]. The complex interplay of the aforementioned mechanisms may lead to oral dysbiosis. A study identifying oral bacterial dysbiosis in patients with CKD [32] reported oral microbiome changes in which *Capnocytophaga, Tannerella, Streptococcus, and Fusobacterium* levels increased with CKD stage. In CKD patients, dental calculus and periodontal disease can be severe even with low levels of biofilm, and the associated salivary disease and dental caries can lead to tooth loss.

Diabetes mellitus and hypertension are well-known risk factors for adverse outcomes of CKD and are also associated with oral health [33,34]. Diabetes mellitus is characterized by a combination of various somatic factors and factors that affect renal function such as advanced glycosylated end-product, pro-renin, cytokine, and nephrin expression, changes in the renin–angiotensin–aldosterone system, and FGF-23 degradation [35]. Hypertension can lead to CKD by pathogenic mechanisms including sodium dysregulation and increased sympathetic nervous system and altered renin-angiotensin-aldosterone system activity [36]. In particular, some of the β-blockers used as antihypertensive agents reduce insulin sensitivity. In addition, calcium channel blockers can increase proteinuria because they increase intraglomerular pressure and dilate the afferent arteriole [37]. Calcium channel blockers are also used in patients undergoing hemodialysis, and these medications, comorbidities, and more can increase the severity of periodontitis, a cause of tooth loss [38]. A recent systematic review and meta-analysis reported a bidirectional association between hypertension and tooth loss [33], and a cohort study found that individuals with diabetes had an increased risk of tooth loss due to periodontal disease [34]. In this study, potential confounding factors such as diabetes mellitus and hypertension were associated with a number of teeth of fewer than 20, and even after adjusting for these factors, the association of CKD and a number of teeth of fewer than 20 was statistically significant. This suggests that diabetes and hypertension may be shared risk factors potentially linking CKD and the number of teeth.

This study analyzed the association between the number of teeth and potential confounding factors. In an age-stratified model, the risk of having fewer than 20 teeth was increased in older adults aged 66 to 79 years. In a previous study [39], 23% of the elderly lost 3 or more teeth over 12 years. Various forms of smoking and oral health are linked [40], and the causal association between smoking and tooth loss and dose-response relationships are well known [40]. In addition, compared with nonsmokers, current smokers showed a 1.97 OR for CKD incidence [41]. Oral health behaviors such as oral care product use, toothbrushing, and annual dental visits were associated with reduced tooth loss [42]. The results of this study support these findings and indicate the importance of maintaining and improving oral health through smoking cessation and oral-related preventive behaviors as age increases.

This study has several limitations. First, because it was a cross-sectional study, it was not possible to confirm a causal association between CKD and the number of teeth. Second, some of the data were collected in the form of self-report questionnaires, which could be biased for recall. Third, the new 2021 CKD-EPI creatinine equation was used to define CKD; thus, the CKD prevalence reported here may differ from those previously reported due to differences in subject characteristics such as race, age, and gender. In addition, Because this was a cross-sectional study, CKD was defined using a single serum creatinine specimen [43,44]. Studies evaluating CKD have used different definitions of CKD, and the results should be interpreted with caution.The KNHANES population is a statistically representative population of Korea in terms of age, gender, geographic location, and social status, and previous studies [45] have shown that the eGFR class concordance between MDRD 2006 and CKD-EPI 2009, and between CKD-EPI 2009 and CKD-EPI 2021 is greater than 99.0%. In addition, the 2021 CKD-EPI equation, which excludes the race variable, has high predictive power and may eliminate health disparities between races [11,46,47]. Fourth, this study adjusted for various comorbidities and oral factors to determine the association between CKD and tooth loss. However, data on factors that may be associated with CKD, such as RAS inhibitors, potassium, and PTH, were not available, and thus, these factors were not investigated in the analysis. Fifth, running analyses on CKD severity may provide insights into mechanistic considerations, but we were unable to do so due to the limited number of patients in the high-risk stratum. Future studies with larger numbers of patients are needed.

However, the KNHANES data were evaluated by trained experts and staff and are highly reliable because sample weights and adjustment were used in the analysis with national data that can represent the entire population.

In summary, This study suggests that CKD is significantly associated with tooth loss. Therefore, when establishing a comprehensive health prevention and management plan for individuals with CKD, it is thought that efforts to link programs to improve oral health will be necessary. Dental professionals should be aware of the impact of various factors including CKD on a decreased number of teeth. In the future, large-scale longitudinal studies adjusted for additional risk factors are needed to clarify the relationship between tooth loss and CKD.

## Data Availability

The datasets generated during and/or analyzed during the current study are available in the Korea National Health and Nutrition Examination Survey repository, https://knhanes.kdca.go.kr/knhanes/sub03/sub03_02_05.do.
